# Evaluation of Anti-Bacterial and Anti-Biofilm Activity of Native Probiotic Strains of* Lactobacillus* Extracts

**DOI:** 10.61186/ibj.4043

**Published:** 2023-12-17

**Authors:** Elmira Karimzadeh Barenji, Shokufeh Beglari, Azar Tahghighi, Parisa Azerang, Mahdi Rohani

**Affiliations:** 1 Department of Biology, Science and Research branch, Islamic Azad University, Tehran, Iran;; 2 Medicinal Chemistry Laboratory, Department of Clinical Research, Pasteur Institute of Iran, Tehran, Iran;; 3 Department of Bacteriology, Pasteur Institute of Iran, Tehran, Iran;; 4 Research Center for Emerging and Reemerging Infectious Diseases, Pasteur Institute of Iran, Tehran, Iran

**Keywords:** Biofilms, Lactobacillus, Drug resistance

## Abstract

**Background:**

Lactic acid bacteria produce various beneficial metabolites, including antimicrobial agents. Owing to the fast-rising antibiotic resistance among pathogenic microbes, scientists are exploring antimicrobials beyond antibiotics. In this study, we examined four *Lactobacillus* strains, namely *L. plantarum *42, *L. brevis *205,* L. rhamnosus *239, and* L. delbrueckii *263, isolated from healthy human microbiota, to evaluate their antibacterial and antifungal activity.

**Methods:**

*Lactobacillus* strains were cultivated, and the conditioned media were obtained. The supernatant was then used to treat pathogenic bacteria and applied to the growth media containing fungal and bacterial strains. Additionally, the supernatant was separated to achieve the organic and aqueous phases. The two phases were then examined in terms of bacterial and fungal growth rates. Disk diffusion and MIC tests were conducted to determine strains with the most growth inhibition potential. Finally, the potent strains identified through the MIC test were tested on the pathogenic microorganisms to assess their effects on the formation of pathogenic biofilms.

**Results:**

The organic phase of *L.* *rhamnosus* 239 extracts exhibited the highest antibacterial and antibiofilm effects, while that of *L. brevis* 205 demonstrated the most effective antifungal impact, with a MIC of 125 µg/mL against *Saccharomyces cerevisiae*.

**Conclusion:**

This study confirms the significant antimicrobial impacts of the LAB strains on pathogenic bacteria and fungi; hence, they could serve as a reliable alternative to antibiotics for a safe and natural protection against pathogenic microorganisms.

## Introduction

In recent decades, medical experts have expressed concerns about the emergence of bacterial antibiotic resistance^[^^1^^]^. O'Neill warned that microbial infections, even mild ones, can lead to severe health problems by 2050, with a potential death toll surpassing that of cancer cases^[^^2^^]^. The frequent alterations of bacterial genetic determinants, coupled with the unregulated and self-administered usage of antibiotics, are crucial factors in the development of antibiotic resistance, posing an imminent threat to human and veterinary healthcare^[^^3^^,^^4^^]^.

Biofilm formation represents a characteristic of microorganisms that confers antibiotic resistance. Biofilm is an assemblage of microbial cells attached to a surface and enclosed in an exopolymer matrix that fosters bacterial virulence and amplifies antibiotic resistance^[^^5^^]^. Compared to planktonic cells, microbial cells within a biofilm exhibit higher levels of resistance to antibiotics^[^^6^^]^, making the elimination of biofilm infections challenging^[^^7^^]^. This resistance is related to the existence of "super resistance cells", which contribute to the regrowth of cells within the biofilm matrix during prolonged antibiotic therapy^[^^8^^]^. *Mycobacterium tuberculosis*,* Acinetobacter baumannii*,* Staphylococcus aureus*, and* P. aeruginosa* are pathogens that develop resistance against antibiotics by applying different resistance mechanisms^[^^9^^]^. Of note, the latter two bacteria have the potential to establish a robust biofilm, which enhances their survival rate^[^^10^^,^^11^^]^. The formation of a biofilm is a crucial strategy for the survival of these microorganisms under stressful conditions, such as those imposed by the host defense system or antibacterial treatments^[^^12^^]^. 

Researchers have attempted to explore alternative natural resources other than antibiotics as novel approaches for treating infections^[^^13^^,^^14^^]^. Of particular interest is the secondary metabolites, which are primarily produced by organisms in an immune-compromised condition and act to protect the host against unfavorable circumstances^[^^15^^]^. Secondary bacterial products, such as organic acids, alcohols, bacteriocins, and hydrogen peroxide, have also captured the attention of researchers^[^^16^^]^. To date, numerous bacterial strains with the potential to synthesize antimicrobial components have been identified^[^^17^^]^. Notably, probiotic bacteria have demonstrated a remarkable capacity for generating diverse antimicrobials^[^^18^^]^. These microorganisms are considered health-friendly and known to have beneficial effects on the body's health when consumed in sufficient amounts^[^^19^^,^^20^^]^. Lactic acid bacteria have gained popularity in the food and cosmetics industries and are commonly employed as preservatives in fermented food products and dairy commodities^[^^21^^,^^22^^]^. These antimicrobial peptides serve as an effective preservative and have a wide range of effectiveness. Bacteriocins as antimicrobial peptides are utilized in *Lactobacillus*-based food products due to their capability in inhibiting the growth of pathogenic bacteria. Lantibiotics, specifically pediocin, have also been observed to suppress a diverse range of Gram-positive bacteria^[^^23^^]^. Therefore, LAB could serve as suitable co-operators in medical therapies; for instance, *Lactobacillus acidophilus* NCFM, produces lactation B with the ability to halt the growth of certain Enterococcus strains^[^^24^^]^. 

Our research stands out for utilizing specific *Lactobacillus* strains obtained from the microbiome of the gastrointestinal tract of healthy individuals. The probiotic effects of these strains have been validated in our prior investigation^[^^25^^]^. Because of their safety and beneficial properties, antimicrobial agents produced by them are considered reliable and appropriate for use in food and medical supplement industries^[^^25^^]^. The aim of the current investigation was to analyze four strains of lactobacilli to produce novel compounds with antimicrobial properties as potential natural substitutes for antibiotics.

## MATERIALS AND METHODS

**Probiotic and pathogen bacterial strains**

*Lactobacillus* strains, including *L. brevis *205,* L. rhamnosus *239,* L. plantarum *42, and* L. delbrueckii *263, isolated from healthy human feces and investigated in our previous study^[25]^, were examined for their antimicrobial and antibiofilm impacts. *E. coli *25922, *B. subtilis *6051, *P. aeruginosa *27853, *S. aureus *25923, *Shigella flexneri *12022,* S. enterica *1231,* Candida albicans *10231, and* Saccharomyces cerevisiae *BY4741 were purchased from the Type Culture Collection of Pasteur Institute of Iran (Tehran). 

**Optimization of the cultural conditions for** ***Lactobacillus*** **strains**

To optimize the cultural conditions and identify suitable media for growth, the *Lactobacillus* strains were inoculated into the commercial MRS broth (Merck, Germany) and a whey culture medium. The culture media, used for the growth of *Lactobacillus* strains, were self-formulated by mixing 10 g each of sucrose and whey powder and 1 L of distilled water. The mixture was heated until all ingredients were dissolved in the water, then autoclaved and divided into conical flasks for bacterial cultivation. The stock culture of the strains was activated by cultivation in 10 mL of MRS broth for 24 h. Afterwards, bacterial cells from MRS broth were seeded on an MRS agar medium and incubated at 37 °C for 24 h. The next day, a few colonies of each bacterial strain were suspended in 5 mL of MRS broth in a conical tube and vortexed. To activate the starter cells, colonies were returned to the incubator for an additional 6-8 h. After removing, the centrifuge tube was left at room temperature for further testing. Fresh growth broth of each bacterial strain was added to conical flasks containing 250 mL of MRS broth and incubated for 24 h. Eight conical flasks containing autoclaved MRS and whey broths (four flasks for each) were prepared individually for each strain. Bacterial strains of the previous section were seeded on MRS and whey broths and incubated in a shaking incubator for four consecutive days. One MRS and one whey flask were taken out daily for four days and analyzed after 24, 48, 72, and 96 h of growth. After these time periods, each conical flask was centrifuged. In brief, the entire content of each conical flask was divided into small samples and centrifuged at 7,552 ×g for 15 min. The supernatants were collected in an autoclaved separatory funnel and kept for the extraction assay.

**Extraction of secondary metabolites from the supernatant**

Extraction assays were performed three times using ethyl acetate. The main steps of the extraction process were carried out the same for the supernatant of the *Lactobacillus* strains in both MRS and whey broth media. The organic and aqueous layers were separated by shaking the separatory funnel loaded individually with MRS and whey broth for five minutes, then allowing it to rest for a few minutes at room temperature. The aqueous phase was filtered using a syringe filter, lyophilized and kept for further investigations. The organic phase was evaporated at 50 °C using a rotary evaporator to determine the final weight of the organic phase. At the end of the experiment, to treat four *Lactobacillus* strains, we utilized four extraction tubes at four times (24, 48, 72, and 96 h) for each MRS and whey broth, totaling eight extracts.

**Antimicrobial assays**

***Well diffusion method***

The well diffusion method was used to determine the antibacterial effect of the cell-free filtered supernatant of the strains on six pathogenic bacterial strains as test strains, which has previously been described with slight modifications^[^^26^^]^. In brief, 100 mg of each extract was dissolved in 1 mL of acetone and left to stand for testing, with a duration of 96 h for bacteria and 72 h for fungi. Pathogenic strains were cultivated on Mueller-Hinton agar for 24 h. The following day, a 0.5 McFarland suspension of the cultures (1.5 × 10^8^ CFU/mL) was prepared, seeded and spread on a Mueller-Hinton agar medium. Sabouraud dextrose agar medium was used for culturing fungal strains. Using a special applicator, eight wells, with a diameter of 6 mm, were created on the agar plates. Each well received 100 µL of the filtrated extract of the 96-h organic phase and incubated at 37 °C overnight. After 24 h, the GIZ around each well was measured with the aid of a ruler. There was no significant difference among the GIZ diameters of the 24, 48, 72, and 96-h extracts. Considering the biomass weight, to evaluate the MIC, we continued the experiments by using the 96-hour extract for bacterial pathogens and the 72-hour extracts for fungal strains, which exhibited the highest extract weight.

***MIC test***

The MIC test was performed separately for the organic and aqueous phases after extracting four *Lactobacillus* strains from MRS and whey cultures, followed by the same steps for each trial. The MIC test was performed according to CLSI instruction (CLSI [NCCLS], 2006; 26:M7-A7), with minor modifications. In brief, standard strains were cultivated on Mueller-Hinton agar and incubated at 37 °C overnight. After 24 h, 0.5 McFarland concentration (1.5 × 10^8^ CFU/mL) was prepared from each strain. In a 96-well microplate, all wells received 100 µL of Mueller-Hinton broth for bacterial strains and Sabouraud dextrose medium for fungal strains, except for the wells in the first row, which received 160 µL of each. Then 40 µL of organic and aqueous phases, obtained from the extraction assay, was added to the first well, individually. Penicillin and itraconazole were added to the well as positive controls and DMSO as a negative control to make a total volume of 200 µL. Thereafter, 100 µL of the content of the first well was serially diluted into the other wells. In the end, 100 µL of the previously prepared 0.5 McFarland suspension of each standard bacterial strains was added to the wells. Microplates were finally incubated at 37 °C for 24 h.

**Antibiofilm assay**

A biofilm assay was performed on *S. aureus* 25923 and *P. aeruginosa *27853 to investigate the antibiofilm activity of the extracts. In brief, *S. aureus* and *P. aeruginosa *were cultivated on Mueller-Hinton agar and incubated at 37 °C overnight. Then 0.5 McFarland suspension of the two bacterial strains (containing 1.5 × 10^8 ^cells) was prepared. The sampling volumes in the 96-well plate were the same those in the MIC test. In the test, we used only Tryptic Soy Broth medium and the four strain extracts with 0.5 McFarland concentration. After incubating the microplates at 37 °C overnight, the contents of each well were discarded and washed three times with sterile phosphate buffer. Microplates were placed inverted at room temperature for 1 h to dry. Thereafter, a TTC dye was used to determine MBIC. A 1% TTC dye solution was prepared using the sterile double distilled water. Then 20 µL of TTC solution was added to each well of the dried microplates, which were then incubated at 37 °C for 15 minutes. Afterward, the dye was removed, rewashed with phosphate buffer and dried at room temperature. Finally, 200 µL of 30% acetic acid was added to each well to extract the dye bound to the bacterial biofilm. The absorbance of the color solution was measured at 595 nm using a microplate reader. 

**Statistical analysis**

The data were analyzed using Graph Pad Prism version 8.0.2.263 using one-way analysis of variance (ANOVA) and t-tests. *P *values less than 0.05 were considered statistically significant.

## RESULTS

**Optimization of cultural conditions in MRS- and whey-based media**

**Fig. 1:**
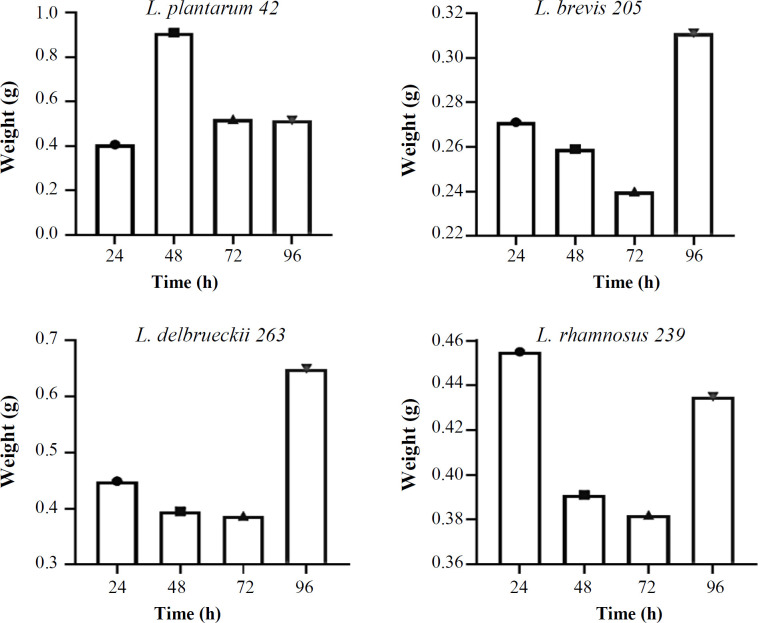
Biomass weight of *Lactobacillus* strains in MRS-based media at different growth times. *L. brevis* 205, *L. delbrueckii* 263, *L. plantarum *42, and *L. rhamnosus* 239 had the highest weight after 96, 96, 48, and 24 h, respectively

**Fig. 2:**
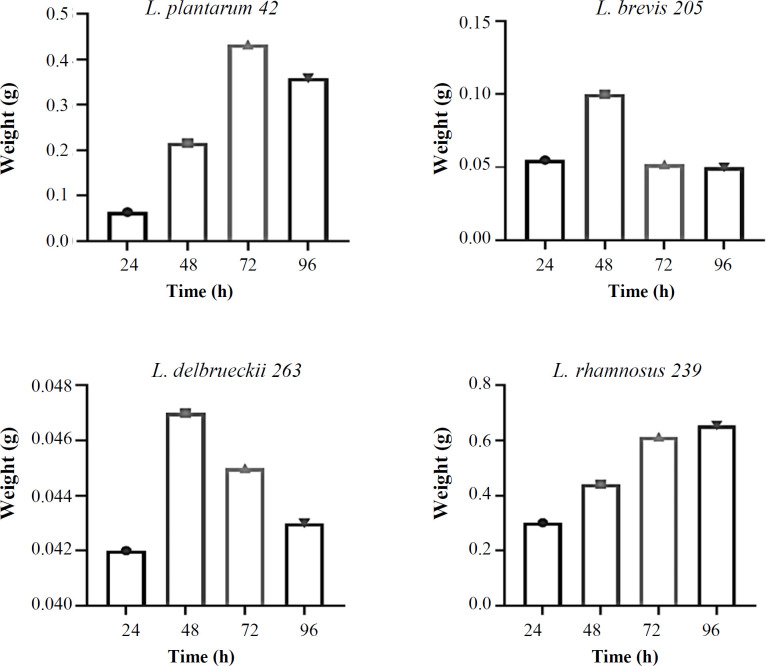
Biomass weight of *Lactobacillus* strains in whey-based media at different growth times. *L. brevis* 205, *L. rhamnosus* 239, *L. delbroeckii* 263, and *L. plantarum* 42 had the highest weight after 48, 96, 48, and 72 h, respectively

**Fig. 3:**
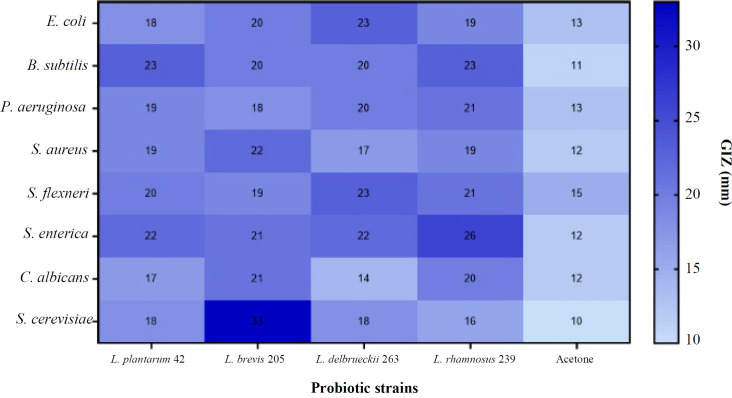
GIZ produced by *Lactobacillus* organic phase against bacterial and fungal strains. *S*. *cerevisiae* showed the widest GIZ.  GIZ is observed in all test strains, confirming the effectiveness of the antimicrobials in the *Lactobacillus* extracts

**Fig. 4:**
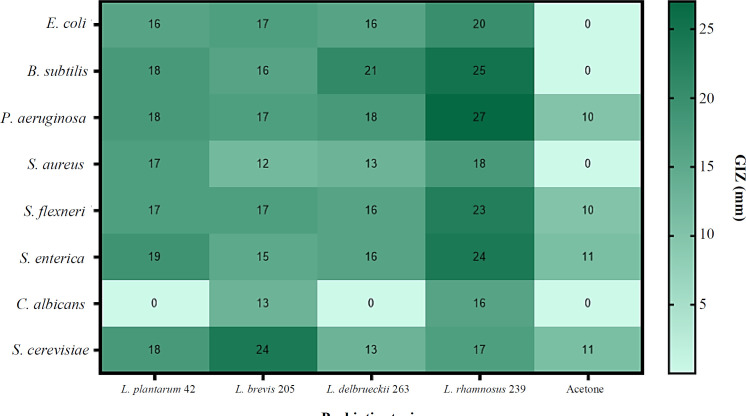
Antimicrobial effect of *Lactobacillus* whey-based media against the test bacteria. The whey-based media were powerfully effective against most bacterial test strains. Acetone did not inhibit the growth of the test strains, except for the *P. aeruginosa *and  *S. enterica,* with a narrow GIZ

The effect of growth time on the biomass weight of *Lactobacillus *strains in MRS- and whey-based media is shown in Figure 1. *Lactobacillus *strains exhibited different biomass production patterns in different time periods. After extracting the supernatant of MRS broth from the strains, it was observed that *L. brevis* 205 and *L. plantarum* 42 had the highest weight after 96 and 48 h, followed by *L. delbrueckii* 263 and *L. rhamnosus* 239 after 96 and 24 h, respectively (Fig. 1). The extracts of whey-based media of the *Lactobacillus* strains at different time points exhibited the highest biomass weight of *L. brevis* 205, *L. rhamnosus* 239, *L. delbrueckii* 263, and *L. plantarum* 42 after 48, 96, 48, and 72 h, respectively (Fig. 2). Comparing the GIZs of *Lactobacillus* strains grown in MRS- and whey-based media for 24, 48, 72, and 96 hours showed minimal discrepancies. Therefore, the 96-hour extract was chosen for subsequent analyses. Figure S1 shows the GIZs produced by *L. brevis* 205 against different bacteria, which were selected from one of the four *Lactobacillus* strains. The inhibitory potential of *Lactobacillus* strains cultivated in MRS-based media exhibited the acceptable inhibitory effect of all extracts on the target strains. Notably, the GIZ was observed in almost all tested bacteria, indicating the ability of the extracts of 72 and 96 h to inhibit the growth of bacterial and fungal strains (Fig. 3). The diameters of the GIZ are detailed in Table 1. Regarding *Lactobacillus* extracts derived from whey broth media, we observed minor variations in the GIZ diameters. Due to the lower mass of whey-based media compared to MRS-based media, only the 72-hour extract of *L. rhamnosus* 239 was chosen for subsequent experiments. Evaluating the inhibitory effects of whey-based media containing *Lactobacillus* strains on the target strains demonstrated that the 72-hour extracts yielded diverse outcomes against the target bacteria. Notably, growth inhibitory effects were observed on *P. aeruginosa*, *S. subtilis*, *E. coli*, *S. enterica*, and *S. flexneri*. A distinct GIZ was observed in the extracts of *L. brevis* 205 for the fungal strains, while smaller GIZs were observed in other samples (Table 1 and Fig. 4)

**MIC test**

**Fig. 5:**
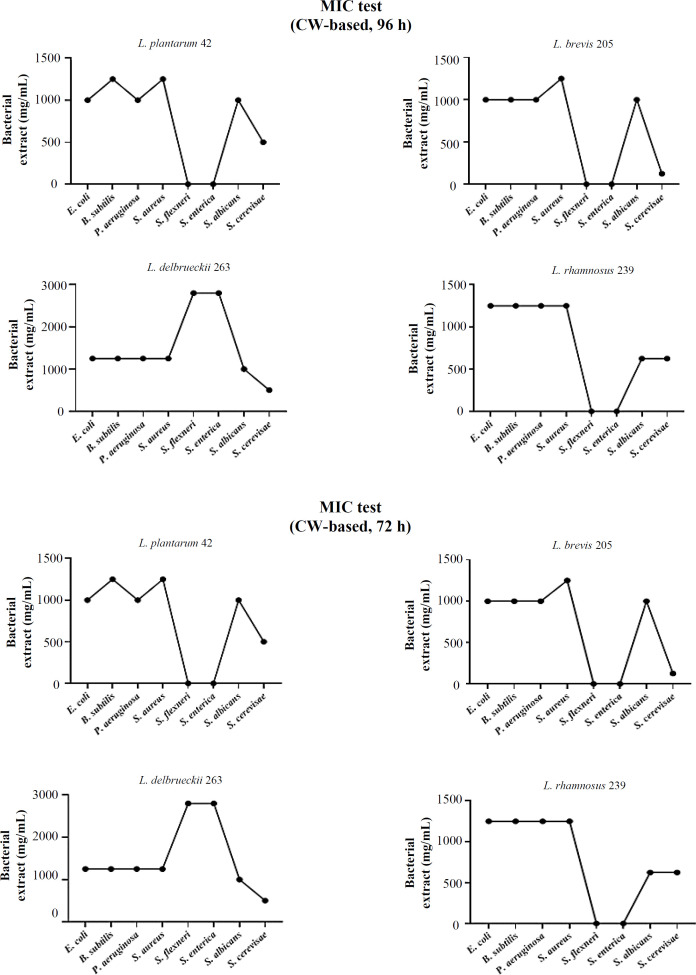
MIC test results for 96-h extracts of MRS and 72-h extracts of whey-based cultures. An acceptable growth inhibition occurred for most of the pathogen bacteria in response to the extracts of the LAB strains, including *L. brevis *205,* L. rhamnosus *239,* L. delbrueckii *263, and *L. plantarum *42

Based on the results of the MIC examination, the extracts of whey- and MRS-based media of *L. brevis* 205 demonstrated significant growth inhibition efficacy against *S. cerevisiae* compared to the other strains examined. This observation was also consistent in *L. brevis* 205, *L. delbrueckii* 263, and *L. rhamnosus* 239. The extracts of all strains, whether derived from MRS or whey, exhibited a significant growth inhibitory effect on the test strains. Figure 5 presents the 96-hour MIC findings for the test bacteria.

**MBIC** **test**

**Fig. 6:**
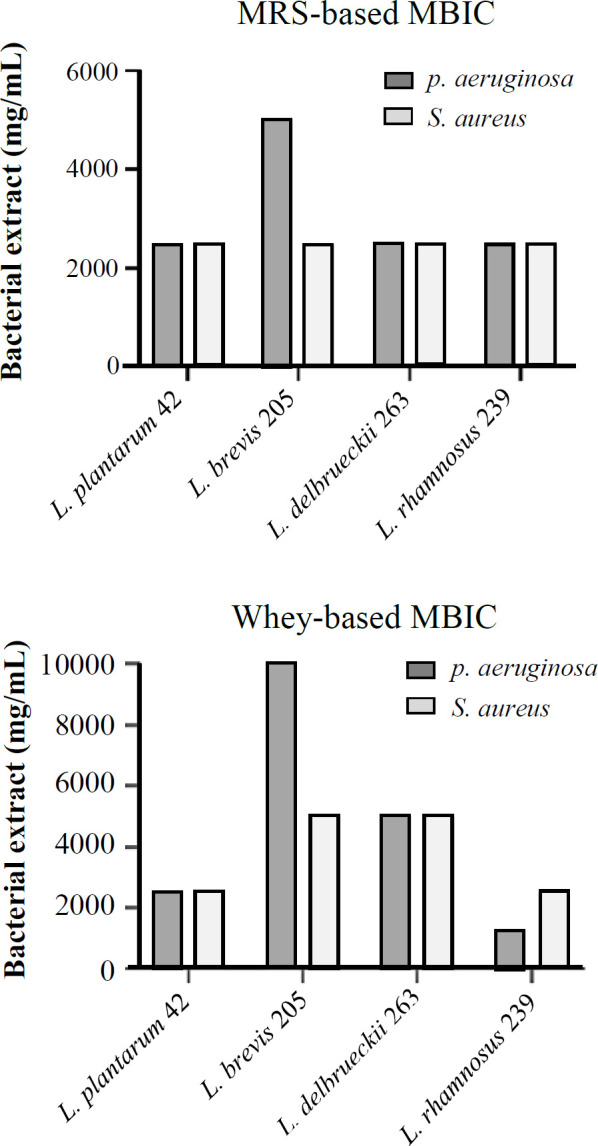
The MBIC test for *P. aeruginosa *and* S. aureus*. Extracts of MRS-based media were more effective than whey-based media on biofilm formation inhibition

The 96-h MRS-based media of *L. plantarum* 42, *L. rhamnosus *239, and* L. delbrueckii *263 prevented the formation of the* S. aureus *biofilm to a significant extent; however, *L. brevis* 205 was relatively ineffective. In whey-based media, extracts of *L. plantarum* 42 and *L. rhamnosus* 239 showed similar inhibitory effects on biofilm formation, while *L. delbrueckii* 263 and *L. brevis* 205 showed significantly less impact on biofilm inhibition compared to the two other strains. The results of MRS-based sections obtained from the culture medium were more effective than the whey-based media in inhibiting biofilm formation. The MBIC test exhibited that *P. aeruginosa*, the same as *L. plantarum *42,* L*. *brevis *205, and *L. rhamnosus* 239, could inhibit biofilm formation. In contrast, *L. delbrueckii* 263 showed less inhibitory performance to biofilm formation (Fig. 6 and Table 2).

## DISCUSSION

Over the course of approximately 90 years of global antibiotic use, the emergence of antibiotic-resistant infections has become increasingly problematic. To solve this problem, the medical experts focused their efforts on limiting bacterial resistance to antibiotics, which has necessitated the search for alternative and efficient substitutes to replace antibiotics^[^^27^^]^. This problem is maximized when pathogenic microbes form a biofilm, resulting in increased antibiotic resistance. Consequently, the symptoms of outbreaks are worsened, detection methods are became costly, and therapies will be complicated, all of which pose a significant threat to human life^[^^9^^]^.

*S. aureus* and *P. aeruginosa* are two of the primary culprits behind bedsores and hospital-acquired infections. The production of a biofilm by these organisms results in increased virulence^[^^12^^]^, prompting a strong demand for novel antimicrobial agents with antibiofilm properties^[^^28^^]^. With the increasing interest in natural metabolites for pharmaceutical applications, biological resources have attracted researcher’s attention for antimicrobial purposes^[^^29^^]^. 

Probiotic bacteria are the most well-known bacterial strains due to their beneficial impacts on human health. LAB protect the body from pathogens by competitively attaching to the host cells and synthesizing a broad range of organic compounds, including organic acids and antimicrobial agents^[^^30^^]^. Probiotic bacteria, due to their safety, permissibility, and non-pathogenic nature, have been identified as a promising source of antimicrobial products that could potentially serve as a substitute for antibiotics^[^^31^^,^^32^^]^.

The present study investigated the antimicrobial effects of microbiota-based LAB by applying their extracts to pathogenic bacterial and fungal strains. Our previous study has confirmed the presence of a bacteriocin gene in our bacterial samples^[^^25^^]^. Herein, we investigated the antimicrobial potential of these bacteria using two different culture media (MRS and cheese whey) in four different growth periods. The results showed that the organic phase of the MRS-based media was much more effective in eliminating the pathogenic test strain and preventing their growth with acceptable potency. Furthermore, the whey-based media had the same inhibitory effects on the growth of the test bacteria; however, the aqueous phase did not have a significant impact on the test strains (data not shown). Compared to the whey-based media, the MRS-based media were more substantial. 

**Table 1:** GIZ of pathogenic test strains in response to *Lactobacillus* 96-h and 72-hour extracts in MRS-based and whey-based media

**Extracts**	**Test strain GIZ (mm)**							
**96-h MRS**	***E. coli***	***B. subtilis***	***P. aeruginosa***	***S. aureus***	***S. flexneri***	***S. enterica***	***C. albicans***	***S. cerevisiae***
*L. plantarum* 42	18	23	19	19	20	22	17	18
*L. brevis* 205	20	20	18	22	19	21	21	33
*L. delbrueckii* 263	23	20	20	17	23	22	14	18
*L. rhamnosus* 239	19	23	21	19	21	26	20	16
Acetone	13	11	13	12	15	12	12	10
**72-h whey **								
*L. plantarum* 42	16	18	18	17	17	19	1	18
*L. brevis* 205	17	16	17	12	17	15	13	24
*L. delbrueckii* 263	16	21	18	13	16	16	1	13
*L. rhamnosus* 239	20	25	27	18	23	24	16	17
Acetone	1	1	10	1	10	11	1	11

The secondary metabolites of a microorganism are generally produced during the stationary phase^[^^33^^]^. According to this fact, we selected the 96- and 72-h extracts of MRS and whey-based media, respectively, to achieve the highest yield. The results showed that the 96-h MRS-based media of *L. plantarum *42,* L. brevis *205, and *L. rhamnosus *239 had a significant antibacterial effect on pathogenic bacteria. *L. plantarum *42, with a GIZ of 22 mm, exhibited a satisfying outcome. Lin and Pan investigated the antimicrobial properties of the supernatant of *L. plantarum* NTU 102. 

Their results revealed that the supernatant had a wide range of antimicrobial effect on both Gram-positive and Gram-negative bacteria, as well as yeasts. Plantaricin, a bacteriocin found in the *L. plantarum* species with a wide variety of structural features, might be a probable contributor to the antimicrobial effects of this strain, particularly against gastrointestinal pathogens. These findings affirm our results, which also emphasize the antimicrobial efficacy of *L. plantarum* 42^[34]^. Our findings revealed superb destructive effect of the 96-h extract of *L. brevis* 205, *C. albicans*,* S. cerevisiae*, and *S. aureus*, with a GIZ of 33 mm. The 96-h extract of *L. rhamnosus* 239 was an effective growth inhibitor of *S. enterica* and *P. aeruginosa*, with GIZs of 21 and 26 mm, respectively. Both MRS- and whey-based extracts of *L. brevis* 205 exhibited the highest fungal inhibitory effect on *S. cerevisiae*, with a MIC of 125.5 µg/mL. The MRS-based medium of *L. rhamnosus* 239 demonstrated a significant antifungal activity, with a MIC of 312.5 µg/mL. 

LAB could be derived from diverse sources, including nature, fermented foods, and microbiota. Numerous surveys have been conducted on *L. plantarum* strains isolated from various sources to evaluate their potential as producers of antimicrobial agents. Li et al. evaluated the resistance of *L. plantarum* strains, obtained from the Chinese fermented food, to hydrogen peroxidase^[^^35^^]^. The findings of their study indicated a correlation between the antioxidative activity of the species and their origin of isolation^[^^35^^]^. Essid et al. investigated the antimicrobial effects of the *L. plantarum* strains isolated from salted meat on *S. aureus* and *P. aeruginosa *and observed that the most inhibitory effect was against *S. aureus*^[^^36^^]^*.* The source of the LAB utilized in the present study was healthy human microbiota, which serves as a highly valuable reservoir for antimicrobial agents.

Microbiota is a complex composition of micro-organisms and primarily protects the human body by engaging in competitive attachment mechanisms against pathogens. These microorganisms utilize a variety of strategies to combat pathogens, including producing peptides with potent antimicrobial properties. This function helps prevent the attachment and proliferation of pathogens, ultimately allowing them to maintain their niche^[^^31^^]^. Therefore, as members of the microbiota, LAB strains in our study were expected to potentially produce antimicrobial agents, which was confirmed by our results.

**Table 2:** MBIC results of the MRS and whey-based media extracts of *Lactobacillus* strains

**Organic extracts**	***P. aeruginosa *** **27853**	***S. aureus *** **25923**
	**Organic layer (µg/mL)**	
*L. plantarum 42*	2500	2500
*L. brevis 205*	5000	2500
*L. delbrueckii 263*	2500	2500
*L. rhamnosus 239*	2500	2500
*L. plantarum 42*	2500	2500
*L. brevis 205*	10000	5000
*L. delbrueckii 263*	5000	5000
*L. rhamnosus 239*	1250	2500
**Control**		
DMSO/DDW	10% v/v	10% v/v

After identifying highly effective strains for the production of antimicrobial agents, the extraction method of these chemicals becomes crucial. So far, in the extraction procedure, researchers have used various chemicals to obtain a perfect supernatant containing antimicrobial agents. Studies have established that the nature and chemical composition of the solvent used for extraction play a decisive role in determining the efficacy and biological activity of the extract. Kim and colleagues used ammonium sulfate to extract a crude bacteriocin from *L. brevis* DF01^[^^37^^]^. De Giani used *n*-butanol with agitation for the organic phase extraction of *L. plantarum* strain PBS067 and then re-suspended the extract in methanol^ [^^38^^]^. Ibrahim and colleagues^[^^39^^] ^extracted the cell-free supernatant of *Lactobacillus helveticus* using ethyl acetate, diethyl ether, and dichloromethane. The ethyl acetate extract showed the most potent destructive effect on all tested pathogens. The most significant impact was observed on *Staphylococcus sciuri*, with a GIZ of 38 mm. Extracts obtained by diethyl ether and dichloromethane had a GIZ of 21 mm. Thus, diethyl ether could be a profitable solvent for extracting unique antimicrobials from bacterial cells^[^^39^^]^, which was successfully used in our research. 

Bacteriocins, such as Nisin, are produced by *Lactococcus lactis* and are widely used as food preservatives. There is a report confirming the acceptable ability of nisin to prevent biofilm formation by foodborne and pathogenic bacteria, such as *S. aureus*^[^^40^^]^*.* Kim et al. isolated *L. brevis* DF01 from kimchi and applied its bacteriocin to *E. coli* KCTC 1039 and *S. typhimurium* KCTC 1925. They showed that the biofilm formation was eliminated after incubation of pathogens with bacteriocin DF01^[^^37^^]^. Further investigation is required to identify the exact antimicrobial agents present in the crude organic phase of the bacterial extracts. The observed antimicrobial activity of the organic phase is a promising indicator for future discoveries of antimicrobial agents. Furthermore, the discovery of metabolic pathways and the synthesis of secondary metabolites are fundamental aspects that are needed to be taken into account in future research. To systematize the field of antimicrobial production, coordination between antimicrobial drug discovery and systems biology is essential.

## CONCLUSION

Antimicrobial agents derived from probiotics present a promising avenue in combating antibiotic resistance and have the potential to enhance the efficacy of antibiotics. Herein, we identified effective antimicrobial compounds and utilized safe, human-compatible strains of *Lactobacillus*. The utilization of producer strains derived from humans may provide non-toxic and beneficial alternatives for the food and pharmaceutical industries. Further exploration is warranted to elucidate the characteristics of the antimicrobial substance synthesized by these probiotic microorganisms.

## DECLARATIONS

### Acknowledgments

 The present research was conducted for the master's thesis in the laboratories of medicinal chemistry and microbiology of the Pasteur Institute of Iran (Tehran). We express our gratitude to Dr. Morvarid Shafiei for her assistance in the project. We declare that we have not used artificial intelligence software in writing this article and have only used online tools such as Typeset and Ginger to make a native English text.

### Ethical approval

All the experimental procedures in this study were approved by the Research Ethics Committee of the Pasteur Institute of Iran, Tehran (ethical code: IR.PII.AEC.1402.016). 

### Consent to participate

Not applicable.

### Consent for publication

All authors reviewed the results and approved the final version of the manuscript.

### Authors’ contributions

EKB: investigations and methodology; SB: review and editing the article and writing the original draft and the data analyzation; AT: conceptualization; PA: supervision and instruction, project administration, and review and editing the article; MR: conceptualization and project administration.

### Data availability

 All relevant data can be found within the manuscript. 

### Competing interests

The authors declare that they have no competing interests. 

### Funding

This research received no specific grant from any funding agency in the public, commercial, or not-for-profit sectors.

### Supplementary information

The online version contains supplementary material.
